# Supplementary material for: A whole-animal phenotypic drug screen identifies suppressors of atherogenic lipoproteins
Source: eLife. 2026 Aug 27;14:RP105314. doi: 10.7554/eLife.105314 (PMC13521566; doi:10.7554/eLife.105314)
Supplement: Supplementary file 2 [file elife-105314-code1.zip › JHDL_validation_result/Relative_data/Medroxyprogesterone.pdf]

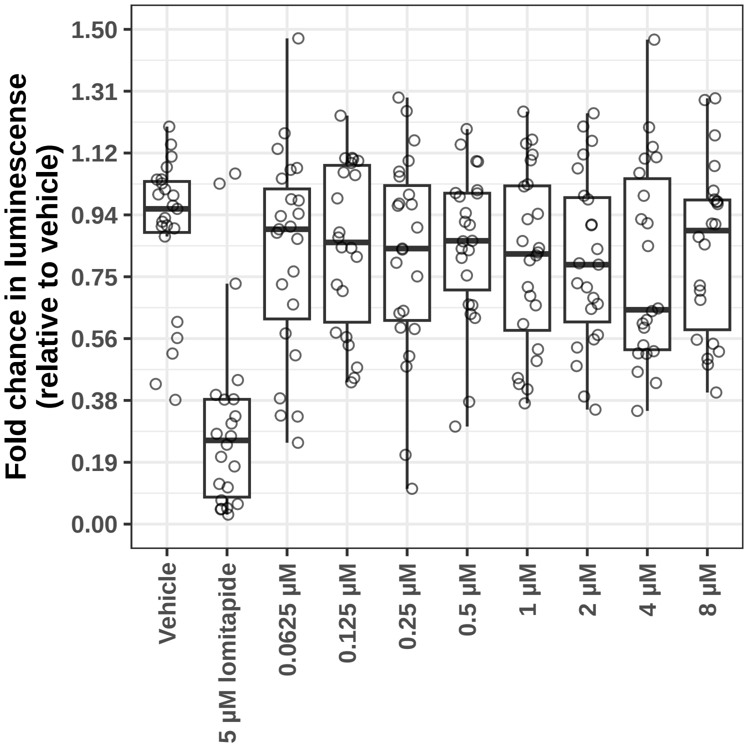

Fold chance in luminescense
(relative to vehicle)
1.50
1.31
1.12
0.94
0.75
0.56
0.38
0.19
0.00
Vehicle
5 µM lomitapide
0.0625 µM
0.125 µM
0.25 µM
0.5 µM
1 µM
2 µM
4 µM
8 µM
